# DNA repair as a shared hallmark in cancer and ageing

**DOI:** 10.1002/1878-0261.13285

**Published:** 2022-07-28

**Authors:** Thomas L. Clarke, Raul Mostoslavsky

**Affiliations:** ^1^ The Massachusetts General Hospital Cancer Center Harvard Medical School Boston MA USA; ^2^ The Broad Institute of Harvard and MIT Cambridge MA USA

**Keywords:** ageing, cancer, DNA damage, genome instability, therapeutics

## Abstract

Increasing evidence demonstrates that DNA damage and genome instability play a crucial role in ageing. Mammalian cells have developed a wide range of complex and well‐orchestrated DNA repair pathways to respond to and resolve many different types of DNA lesions that occur from exogenous and endogenous sources. Defects in these repair pathways lead to accelerated or premature ageing syndromes and increase the likelihood of cancer development. Understanding the fundamental mechanisms of DNA repair will help develop novel strategies to treat ageing‐related diseases. Here, we revisit the processes involved in DNA damage repair and how these can contribute to diseases, including ageing and cancer. We also review recent mechanistic insights into DNA repair and discuss how these insights are being used to develop novel therapeutic strategies for treating human disease. We discuss the use of PARP inhibitors in the clinic for the treatment of breast and ovarian cancer and the challenges associated with acquired drug resistance. Finally, we discuss how DNA repair pathway‐targeted therapeutics are moving beyond PARP inhibition in the search for ever more innovative and efficacious cancer therapies.

## Graphical abstract

In this review, we revisit the processes involved in DNA damage repair and how these can contribute to diseases, including ageing and cancer. We also review recent mechanistic insights into DNA repair and discuss how these insights are being used to develop novel therapeutic strategies for treating human disease.

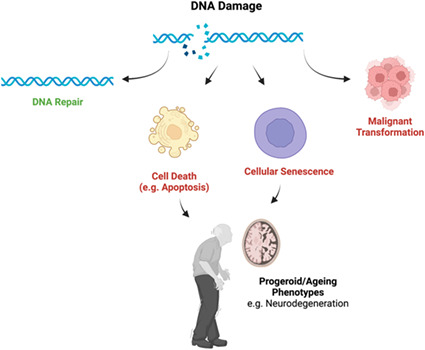

**Abbreviations**
AIF‐1: apoptosis‐inducing factor‐1
Alt‐EJ: alternative end joining
ATM: ataxia telangiectasia mutated
ATR: ataxia telangiectasia and Rad3 related
BER: base‐excision repair
DDR: DNA damage response
DNA‐PK: DNA‐protein kinase
DSBs: DNA double‐strand breaks
FA: Fanconi anemia
HDAC: histone deacetylase
HR: homologous recombination
ICL: interstrand crosslink
IR: ionizing radiation
KO: knock‐out
MMR: mismatch repair
MRN: Mre11‐Rad50‐NBS1
MSI: microsatellite instability
NER: nucleotide excision repair
NHEJ: nonhomologous end joining
PARP: poly ADP‐ribose polymerase
PTMs: post‐translational modifications
RPA: replication protein A
SASP: senescence‐associated secretory phenotype
TOPBP1: DNA topoisomerase 2‐binding protein 1
UV: ultraviolet

## 1 Introduction

Ageing is a complex process, resulting in organismal decline that is characterized at the biological level by the accumulation of extensive molecular and cellular damage [1]. With improvements in modern medicine, epidemiology, and the relative rise in global living standards, the risk of premature death has dramatically decreased in the past century, leading to an increase in life expectancy across most of the world and an ageing human population [2]. The exact causes of ageing are still unclear but given the clear correlation between the accumulation of genetic mutations with increased age, and the impact that this has across practically all tissues, it is tempting to speculate that one major principle of ageing is damage to our genetic information in the form of DNA damage.

Maintaining genome stability is essential for preventing premature ageing and disease. Mutations in DNA repair enzymes result in human syndromes of premature ageing [3] and genomic instability is a fundamental hallmark of cancer [4]. In recent years, the importance of how DNA is packaged within our cells has become apparent, with a plethora of studies linking DNA structure and organization to genome instability and ageing. Importantly, several signaling pathways and molecular processes have also been linked to organismal ageing, including insulin signaling, mTOR signaling, cellular senescence, telomere shortening, and the activity of sirtuins, as we discuss in this review. Indeed, many of these are actively being pursued for purportedly ‘anti‐ageing’ therapies, which have been extensively reviewed elsewhere [5, 6, 7, 8].

In this review, we explore the importance of maintaining genome stability and discuss recent advances in the literature that link this fundamental biological process to mechanisms of ageing and the pathology of ageing‐related diseases. In addition, we discuss recent advances in targeting the cellular pathways that maintain genome integrity for therapeutic purposes to treat age‐related diseases, most notably cancer.

## 2 Chromatin, DNA damage, and ageing

**Fig. 1:**
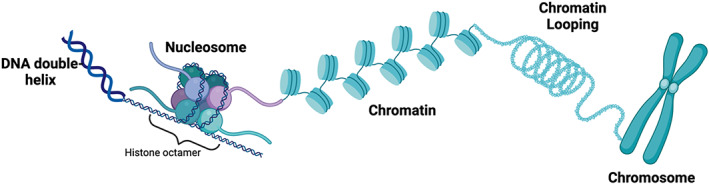
Nucleosome and chromatin structure. Chromatin is made up of nucleosomes, which consist of 147 bp of DNA wrapped around an octamer of histones. Each octamer consists of two molecules of each of the core histones, H2A, H2B, H3 and H4. H3 and H4 form a central tetramer (H3‐H4)_2_, which is flanked by H2A‐H2B dimers. Nucleosomes are repeated structures that are compacted into chromatin. Increasingly condensed chromatin is organized into chromosomes. Figure created using Biorender. [Colour figure can be viewed at wileyonlinelibrary.com]

**Fig. 2:**
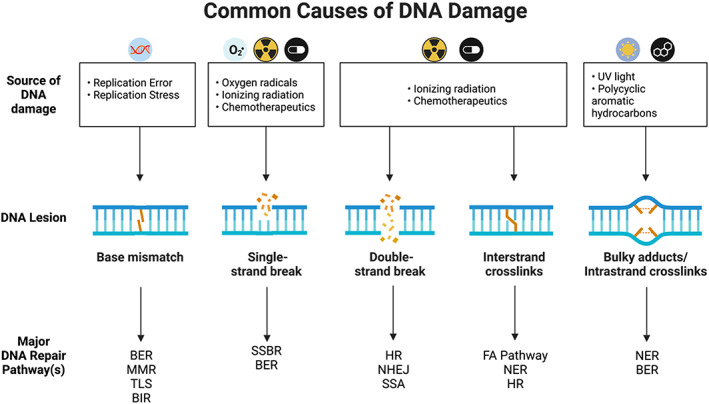
Common causes of DNA damage. Different sources of DNA damage result in distinct DNA lesions, which activate the DDR and a range of DNA repair pathways that repair specific types of DNA damage. BIR, break‐induced recombination; SSBR, single‐strand break repair; TLS, translesion synthesis. Figure created using a Biorender template. [Colour figure can be viewed at wileyonlinelibrary.com]

DNA is packaged into chromatin, the basic unit of which is the nucleosome. Nucleosomes consist of DNA wrapped around an octamer of histones (see Fig. 1) [9]. These histones are targeted by various post‐translational modifications (PTMs), such as methylation, acetylation, SUMOylation, ubiquitylation, ADP ribosylation of lysine residues, and the phosphorylation of serine and threonine residues [2]. These modifications regulate a range of biological processes, including DNA transcription, DNA replication, and DNA damage repair [2, 3, 10]. Different forms of DNA damage, their causes, and the pathways that repair them are shown in Fig. 2.

Interestingly, histone modifications have been shown to alter with age. Histone modifications and DNA methylation have important regulatory roles in chromatin remodeling, and thus in the regulation of transcriptional programs. As such, changes in these epigenetic marks could have wide‐ranging effects on transcriptional programs across tissues. This rationale largely underpins the theory of the ‘epigenetic clock’, in which epigenetic modifications, most notably DNA methylation at specific CpG sites, can be used to infer biological age [11]. Indeed, several studies have reported correlations between DNA methylation status and chronological age [12]. In this context, Fransquet et al. [13] recently performed a meta‐analysis of these correlations in human patients, and whilst they did not find a definitive link between DNA methylation and ageing‐related disease *per se*, they did find a relationship between epigenetic age and increased risk of mortality. When considering changes in the context of ‘the epigenetic clock’, it is important to recognize that these changes are largely assessed at the level of bulk DNA, implying that the CpG methylation observed, for example, would be the same across all cell types. However, there is stochasticity between these cells, especially for methylation status, which has been associated with incomplete restoration of chromatin status following DNA repair [14]. Indeed, a recent study demonstrated an accumulation of stochastic DNA methylation changes in aged mouse muscle stem cells, which was associated with impaired transcriptional networks in these aged cells. This was postulated to be a potential mechanism driving the ‘epigenetic clock’ [15].

A classic example of the importance of epigenetic modulators in genome stability and ageing is the sirtuin family of proteins. Guarente and colleagues were the first to report the Sir family as being key regulators of lifespan and cellular ageing [16]. *Sir2* is a deacetylase that requires the energetic intermediate NAD+ as a cofactor [17]. It is highly conserved across species from archaea to humans, and in mice, *Sir2* homologues have been shown to play a key role in longevity [18, 19, 20]. The phenylpropanoid resveratrol was shown to improve the lifespan of mice on a high‐calorie diet, in a sir2‐dependent manner [20]. However, this remains a somewhat controversial finding for several reasons. Firstly, Fabrizio et al. [21] demonstrated that *Sir2* deletion was able to increase lifespan in a yeast model, which directly contradicts the premise that *Sir2* is a key longevity gene. In addition, there are conflicting reports in the literature as to whether resveratrol is a direct activator of the sirtuin family of proteins [22]. For example, Kaeberlein et al. [23] demonstrated that resveratrol does activate *Sir2* and human Sirtuin 1 (Sirt1) *in vitro*; however, this activation is dependent on a nonphysiological fluorophore. Moreover, consistent with the dependency on a nonphysiological fluorophore, resveratrol had no impact on the lifespan of yeast *in vivo* [23]. Additional work has also demonstrated that whole body overexpression of *Sirt1* in mice, while improving metabolism, does not impact longevity [24]. On the other hand, Satoh et al. [25] showed that, in brain‐specific *Sirt1*‐overexpressing transgenic mice, these mice demonstrated significant lifespan extension in both males and females. It is therefore possible that the impact of Sirtuin expression on lifespan is dependent on expression levels in specific tissues, and this nuance is one possible reason for the complex and often conflicting literature in this field. The anti‐ageing properties of resveratrol are still heavily debated including its precise mechanisms of action. A more recent report adds additional concerns with the use of resveratrol as an anti‐ageing supplement in humans, demonstrating that resveratrol causes genome instability in human cells, indicating the need for caution in the use of resveratrol as an anti‐ageing therapy in humans [26, 27].

To date, seven mammalian Sirtuins have been discovered, *Sirt1‐7*, all of which are implicated in a wide range of biological processes, including transcriptional regulation, DNA repair, metabolism, mitochondrial homeostasis, and cell‐cycle regulation. For more information on the roles of mammalian Sirtuins, we direct readers to these recent reviews [28, 29].

**Fig. 3:**
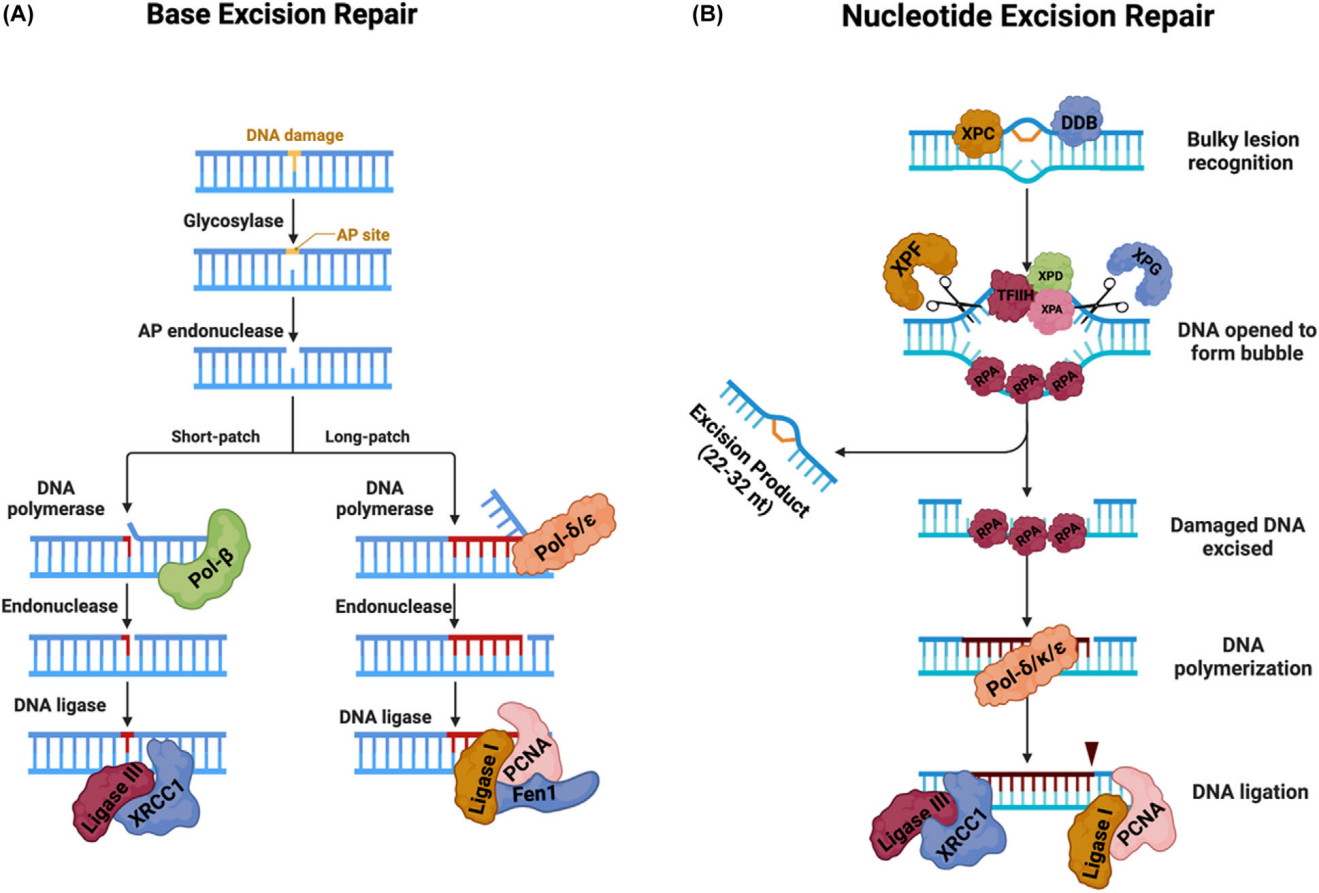
Base‐excision repair and nucleotide excision repairNER pathways. (A) In BER, damaged base(s) are recognized by DNA glycosylases that generate a apurinic/apyrimidinic (AP) damage site [40, 41]. The AP endonuclease (AP‐1) recognizes this AP site and hydrolyzes the DNA back bone to form a single‐strand DNA break (SSB), which is stabilized by PARP1 [42, 43]. PARP1 can recruit several repair factors, including polymerase‐beta (Pol‐(β) [44, 45, 46]. There are two main types of BER, SP‐BER, which repairs single damaged bases, or LP‐BER, which repairs small, damaged stretches of DNA. In SP‐BER, PARP1 recruits polymerase‐beta (Pol‐β) [46], which fills in the gap at the SSB together with DNA Ligase III and XRCC1 [47]. In LP‐BER, 2‐8 nucleotides are synthesized by DNA polymerase δ/ε (Polδ/ε), which then, together with PCNA, FEN1, and DNA Ligase I complete the long‐patch repair of the SSB [48]. (B) In NER damage, the DNA lesion is recognized by XPC‐Rad23B or UV‐DDB [49, 50]. The TFIIH complex interacts with XPC‐Rad23B at the lesion to open up the DNA. This enables the XPD protein to travel along the DNA and stall at the lesion. This stalling creates a binding site for the preincision complex, consisting of XPA‐RPA‐XPG. ERCC1/XPF interacts with XPA to catalyze the 5′ incision of the DNA lesion. Next, DNA is synthesized by Pol δ, Pol κ, or Pol ε, followed by the 3′ incision by XPG. Finally, the remaining nick is sealed by XRCC1/Ligase III‐α or DNA ligase‐1 [49, 50]. Figure created using a Biorender template. [Colour figure can be viewed at wileyonlinelibrary.com]

Sirt6 has histone deacetylase (HDAC) activity, and it has key roles in DNA repair, cancer, and progeroid/ageing phenotypes. Its deletion in mice impairs the base‐excision DNA repair pathway (see Fig. 3A), resulting in increased genome instability in these mice [30]. *Sirt6* knockout mice also exhibit other severe phenotypes, including loss of subcutaneous fat, lymphopenia, and severe metabolic defects—phenotypes that overlap with degenerative processes commonly seen in ageing [30]. Importantly, opposing the phenotype of the KO mice, *Sirt6* overexpressing transgenic mice exhibited increased lifespan [31]. More recent work has shown that Sirt6 is also important in DNA double‐strand break (DSB) repair (see Fig. 2 and Box 1). Indeed, Sirt6 has been shown to be involved in the rapid recruitment of the SNF2H chromatin remodeling factor, which is important for altering chromatin structure to facilitate the repair of damaged DNA [32]. Sirt6 is also described as being a direct sensor of DNA DSBs, solidifying its role as a key guardian of genome stability maintenance in mammalian cells [33]. Additional work has also demonstrated that Sirt6 can mediate the repair of DNA DSBs via both homologous recombination (HR) and nonhomologous end joining (NHEJ), during oxidative stress, via regulation of PARP1 poly‐ADP‐ribosylase activity [34]. Follow‐up studies of Sirt6's involvement in metabolism have also uncovered that the lethal hypoglycemic phenotype seen in *Sirt6* KO mice is driven by the Sirt6‐mediated regulation of glucose homeostasis. This regulation is mediated by Sirt6's H3K9 deacetylase activity, which controls the expression of glycolytic genes [35]. More recently, its metabolic regulatory functions have been shown to be important in cancer as well, with Sirt6 functioning as a tumor suppressor in mouse models and human patients in both colorectal and pancreatic adenocarcinoma [36, 37]. In a parallel comparative study, unique variations in *Sirt6's* DNA sequence were identified between species that changed its enzymatic activity; increased Sirt6 activity correlated with increased lifespan among murine species, a phenotype attributed to increased protection from DNA damage [38]. More recent studies in human cells have also demonstrated that mutations in Sirt6 that affect the protein's enzymatic activity result in perinatal lethality because of a failure to suppress pluripotent gene expression [29, 39]. Together, these studies describe a multitude of roles for Sirt6 across a wide range of biological processes, with important implications for human disease, many of which are associated with ageing.

Box 1 **DNA double‐strand break repair**

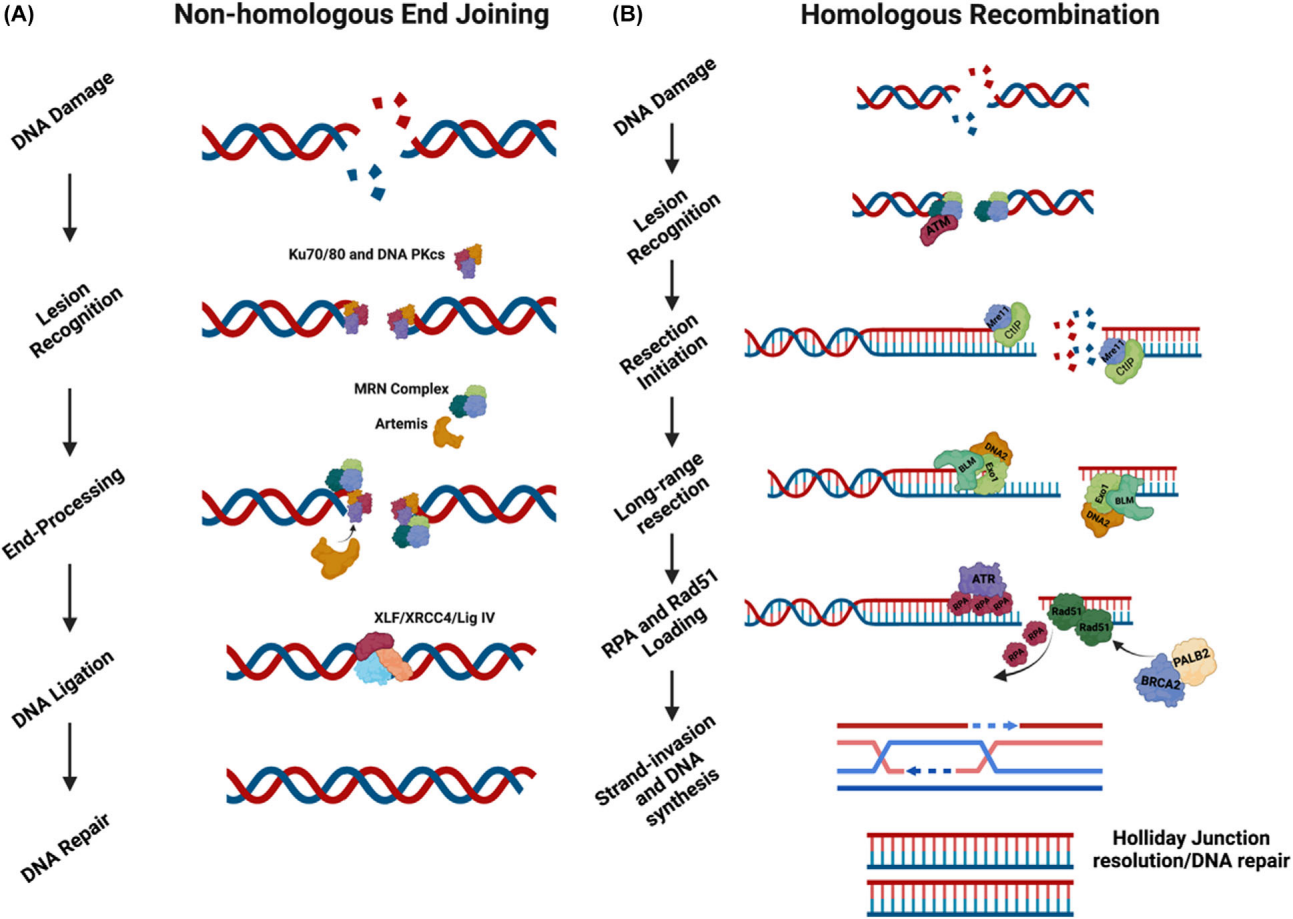

(A) In NHEJ, broken DNA DSB ends are ligated with minimal processing. DSBs are recognized by DNA‐binding proteins, such as the MRN complex, PARP, or the Ku70/Ku80 hetero dimer. Upon competitive binding of Ku70/Ku80 to the break site, the broken DNA ends are tethered in close proximity to each other. The DSB repair protein, 53BP1, in complex with Rev7, RIF1, and the Shieldin complex protects the DNA ends from nucleolytic processing [51, 52, 53]. 53BP1 binds to DNA via both H4K20me2 and H2AK15ub histone modifications, co‐ordinated by the RNF8/RNF168 E3‐ubiquitin ligases [54, 55, 56, 57]. At the break site, 53BP1 acts as a barrier to resection, promoting DNA end ligation by DNA ligase IV, and facilitating the repair of the DSB [58, 59]. (B) In HR, DNA end resection is a complex, cell‐cycle‐regulated process involving the removal of the 53BP1, Rev7, RIF‐1, and Shieldin complex from the DSB site. Once this complex is removed, DNA end resection is initiated by Mre11 and CtIP, which generate a small section of single‐stranded (ss)DNA of ~ 50–100 nucleotides in length, to which the DNA helicases BLM and WRN, and the nucleases DNA2 and Exo1 bind. BLM and WRN help to unwind the DNA double helix, and DNA2 and Exo1 then digest one of the DNA strands to produce a long stretch of ssDNA [60]. How long‐range DNA end resection is mediated by the DNA helicases and nucleases, and how these enzymes are regulated remains under investigation. RPA binds and stabilizes the ssDNA produced by DNA resection and acts as a scaffold for the PALB2‐ and BRCA2‐dependent loading of the DNA recombinase, Rad51 [61, 62, 63]. Rad51 then promotes homology searching and template strand invasion, resulting in error‐free DNA repair by HR [60]. Figure created using Biorender.

In recent years, the importance of epigenetics, beyond DNA methylation and HDACs, has been established across a range of diseases. For example, nonmutational epigenetic reprogramming, such as epigenetically regulated changes in gene expression, is now included as an emerging and enabling hallmark of cancer [64]. The epigenetic regulation of gene expression is also a well‐established regulator of a range of fundamental biological processes, including embryonic development, cellular differentiation, and organogenesis [65, 66]. As described above, altered epigenetic modifications are also associated with cancer development and malignant progression [36, 37, 64]. Indeed, there is mounting evidence of how the tumor microenvironment (including processes such as hypoxia and metabolic change) can directly influence the epigenome [67, 68]. With this in mind, there is an increased interest in targeting the epigenome for cancer therapy, and many inhibitors of epigenetic regulators (KDM4A, EZH2, LSD1, PRMT5) are in preclinical and early phase clinical trials [69].

## 3 DNA repair pathways

DNA can be damaged by numerous exogenous and endogenous sources, resulting in many different DNA lesions (see Fig. 2). Indeed, it is estimated that mammalian cells experience as many as 10^5^ lesions per day [70, 71, 72, 73, 74, 75], most of which are resolved efficiently. However, some lesions escape detection by cellular machinery, are repaired too late, are incorrectly repaired, or are irreparable. The importance of DNA repair for preventing neoplasia is highlighted by the many important dual‐functional roles played by components of the DNA repair machinery in cellular processes, such as cell‐cycle regulation, chromatin remodeling, and ageing [42, 43, 44, 76].

Over time and across species, it is now clear that DNA lesions accumulate, eventually leading to increased genome instability. As such, genome instability is now widely considered to be a hallmark of ageing [77, 78]. Endogenous sources of DNA damage include hydrolysis, oxidation, alkylation, mismatch of DNA bases, inter and intra‐strand crosslinks, and abnormal intermediary structures that can occur through normal physiological processes, including metabolism and DNA replication [79]. Exogenous sources of DNA damage include ionizing radiation (IR) (for example, from X‐rays or radon exposure), ultraviolet (UV) radiation, and various chemical agents, such as platinum‐based chemotherapeutic agents. To repair such lesions, mammalian cells have developed a complex and coordinated network of signaling pathways that can recognize, respond, and repair DNA damage. These processes are collectively known as the DNA damage response (DDR), and they respond to a plethora of different DNA lesions (Fig. 2) [73, 74]. Once the DDR recognizes DNA damage, it initiates cell‐cycle checkpoints, which pause the cell cycle, providing sufficient time for cells to repair the DNA damage before proceeding to cell division. This key process prevents the accumulation of genome instability and/or the loss of genetic material between cell generations. Failure to repair DNA damage can have major consequences for the fidelity of the genome and for overall cell survival. It is widely acknowledged that the failure to repair damaged DNA accurately and efficiently can result in major outcomes, including cell death and cell senescence, which contribute to ageing, and mutation‐inducing cellular transformation, processes that can all lead to cancer development [64]. In this section, we discuss the key mammalian DNA repair pathways and their contributions to ageing and cancer.

### 3.1 Nucleotide excision repair

UV radiation is one of the most common causes of DNA damage and is frequently associated with ageing. UV exposure causes bulky, helix‐distorting lesions that are repaired by the DNA nucleotide excision repair (NER) pathway. NER is a multi‐step process that involves crosstalk between PTMs, resulting in dynamic chromatin changes that facilitate the removal and patching of bulky DNA lesions (see Fig. 3B) [70]. In eukaryotes, NER is categorized into global genomic NER (GG‐NER) and transcription‐coupled NER (TC‐NER). GG‐NER is crucially important for the repair of UV‐associated DNA lesions and for preventing tumorigenesis [80]. Germline mutations that cause NER defects, such as those seen in individuals with Xeroderma Pigmentosum, are associated with hypersensitivity to sun exposure, a significantly increased risk of developing skin cancer, and several progeroid syndromes, as described in more detail below [81, 82].

### 3.2 Base‐excision repair

Base‐excision repair (BER) is utilized in response to endogenous DNA damage, such as oxidative damage, deamination, or alkylation, and particularly to damage that distorts the DNA double helix. There are two main types of BER, short‐patch BER (SP‐BER) and long‐patch BER (LP‐BER) (see Fig. 3A). The number of damaged bases dictates the type of BER pathway that is used. In recent years, it has become clear that BER is a key DNA repair pathway utilized by cancer cells to overcome oxidative DNA damage [83]. Because of this, the inhibition of key components of the BER pathway has been proposed as a therapeutic strategy for treating cancers, including breast and ovarian cancers [83, 84, 85].

### 3.3 Mismatch repair

DNA synthesis at DNA replication forks is not entirely error‐free. Indeed, the frequency of errors by eukaryotic DNA polymerases is estimated to occur at a rate of one error for every 10^5^ nucleotides, resulting in an estimated 100 000 mismatch errors per S phase in cells [86]. Mismatch repair (MMR) corrects spontaneous base mispairing, and small insertions and deletions (indels) that are commonly generated during DNA replication. As DNA polymerases have built‐in proofreading mechanisms, the MMR DNA repair pathway offers a second line of defense against these replication errors. The MMR machinery consists of eight genes in humans (*MSH2, MSH3, MSH5, MSH6, MLH1, MLH2, MLH3, MLH4*), and their primary function in MMR involves lesion recognition, repair initiation, lesion excision, and DNA re‐synthesis. We refer readers to these excellent review articles for more detailed mechanistic explanations of the MMR pathway and its clinical importance [87, 88]. Importantly, defects in proteins of the MMR pathway result in microsatellite instability (MSI), which is now understood to be a key feature in cancer, and in particular colorectal cancer. Indeed, MSI is reported in as many as 15% of sporadic colorectal cancers [89, 90].

### 3.4 Interstrand cross‐link repair

Interstrand crosslinks (ICLs) are extremely cytotoxic DNA lesions that covalently crosslink the two strands of double‐stranded (ds) DNA. Alkylating agents and alkylating chemotherapeutic agents, such as Cisplatin and Mitomycin C, can induce ICLs [91]. Indeed, one reason why Cisplatin and other alkylating chemotherapeutic agents, such as Carboplatin and Oxaliplatin, are effective chemotherapeutics is that the ICLs they induce catastrophically impair critical DNA processes, including DNA transcription and replication [73, 91]. However, these alkylating chemotherapeutic agents do not discriminate between healthy and cancer cells and induce extreme toxicity in patients. To counteract ICLs, cells have developed a specialist ICL repair pathway. Defects in this pathway cause severe chromosome instability syndromes, such as Fanconi Anemia. Fanconi Anemia is one of the best‐known human syndromes associated with defects in ICL repair that is characterized by genome instability, aplastic anemia, bone marrow failure, and cancers, including acute myeloid leukemia [92].

When ICLs occur in the G1 phase, cells heavily depend on the NER pathway to incise and repair the ICL. Indeed, defects in the NER genes that resolve ICLs in the G0/G1 phase result in rare autosomal recessive diseases, including Xeroderma Pigmentosum and Cockayne Syndrome. In S phase cells, ICLs induce DNA DSBs, which are repaired by HR (see Box 1). Cells defective in HR‐mediated repair (for example, those with mutations in *BRCA2*, *PALB2*, or *RAD51* paralogs) exhibit severe hypersensitivity to ICL‐inducing agents [92]. Indeed, many Fanconi Anemia (FA)‐associated genes are important for coordinating HR at stalled or collapsed DNA replication forks [92, 93]. ICLs are detected by the UHRF1 protein in coordination with the FANCM‐FAAP24‐MHF complex, which binds to stalled replication forks at the ICL site. This complex recruits the FA core complex, which consists of ten proteins (FANCA, FANCB, FANCC, FANCE, FANCF, FANCG, FANCL, FAAP100, FAAP20, and FAAP24), and the BLM‐Top3α‐RMI complex [92, 94, 95]. This complex facilitates the activation of the E3 ligase activity of the FA core complex at the stalled replication fork, resulting in the monoubiquitination of FANCD2 and FANCI. The monoubiquitination of FANCD2 and FANCI leads to the recruitment of SLX4/FANCP, which acts as a scaffold protein for the structure‐specific endonucleases Mus81, SLX1, XPF/ERCC4/FANCQ. These endonucleases are licensed to carry out controlled ICL excision, generating a DNA DSB that is repaired by HR [92, 94, 95].

### 3.5 Replicative stress

Replication stress is a genomic emergency for the cell and is loosely defined as anything that impedes or slows the progression of the replication machinery and/or DNA synthesis [96]. Failure to resolve replication stress accurately and efficiently can be catastrophic, resulting in extensive genome instability and cellular toxicity. Indeed, replication stress is implicated in the pathogenesis of several different diseases including cancer and progeroid syndromes [97]. There are many sources of replication stress, and we refer readers to recent reviews on this topic [96, 97, 98]. In brief, causes of replication stress include unrepaired DNA lesions, such as gaps and nicks in ssDNA, and more complex lesions, such as ICLs caused by exogenous agents (as discussed above) [94]. Additional causes of replication stress include misincorporation of ribonucleotides [99], the formation of unusual DNA structures, such as G quadruplexes [100], and the exhaustion of essential replication factors, such as nucleotides; indeed, nucleotide depletion is observed following treatment with the replication stress‐inducing agent, hydroxyurea [101, 102].

Mammalian cells have developed a specialized replication stress response pathway to alleviate the consequences of impaired DNA replication, largely mediated by the ATR (Ataxia Telangiectasia and Rad3‐related) kinase. Cells that lack ATR activity are exceptionally sensitive to agents that impair DNA replication, and the complete absence of ATR results in early embryonic lethality in mice [103, 104]. ATR is a member of the PIKK (Phosphatidyl 3‐kinase‐related kinases) family, which also includes ATM (Ataxia Telangiectasia Mutated) and DNA‐PK (DNA Protein Kinase) [105]. The replication stress response is triggered by the presence of unusual DNA structures, such as abnormally large stretches of ssDNA [96] caused by the uncoupling of the replication complex from the replicative helicase [106]. These large stretches of ssDNA are recognized and bound by the heterotrimeric complex RPA (Replication Protein A; RPA1, RPA2, RPA3), which stabilizes the ssDNA. The resulting RPA‐ssDNA complex then serves as a platform for the recruitment of the ATR‐ATRIP complex [107, 108]. ATRIP interacts directly with the RPA‐ssDNA complex, facilitating its localization to the replication fork [108] and orchestrates the recruitment, binding, and activation of the Rad17‐RFC2‐5 clamp loader, which recruits the 9‐1‐1 complex [109]. The 9‐1‐1 complex is bound to topoisomerase 2‐binding protein 1 (TopBP1), a key DNA repair protein, and so recruits TopBP1 to the stalled replication fork. TopBP1 then activates the ATR‐ATRIP complex by interacting with ATRIP, resulting in ATR kinase activity and the subsequent phosphorylation of the downstream ATR substrate, Chk1 [110, 111, 112]. The subsequent Chk1‐mediated phosphorylation of Cdc25, a phosphatase that functions to activate CDKs by removing inhibitory phosphate groups [113], leads to Cdc25 being sequestered in the cytoplasm by 14‐3‐3 proteins [114]. This reduces CDK activity, leading to cell‐cycle arrest by preventing mitotic entry [115]. ATR activation also helps to protect the stalled replication fork, via the phosphorylation of SMARCAL1, which inhibits fork regression [116], and by helping to facilitate the recruitment of the recombinase Rad51, which protects stalled replication forks from nuclease‐mediated digestion [117, 118, 119]. This ultimately stabilizes the replication fork, preventing fork collapse and DNA double‐strand break formation, thereby enabling the completion of DNA replication and the maintenance of genomic integrity.

### 3.6 DNA double‐strand break repair

**Fig. 4:**
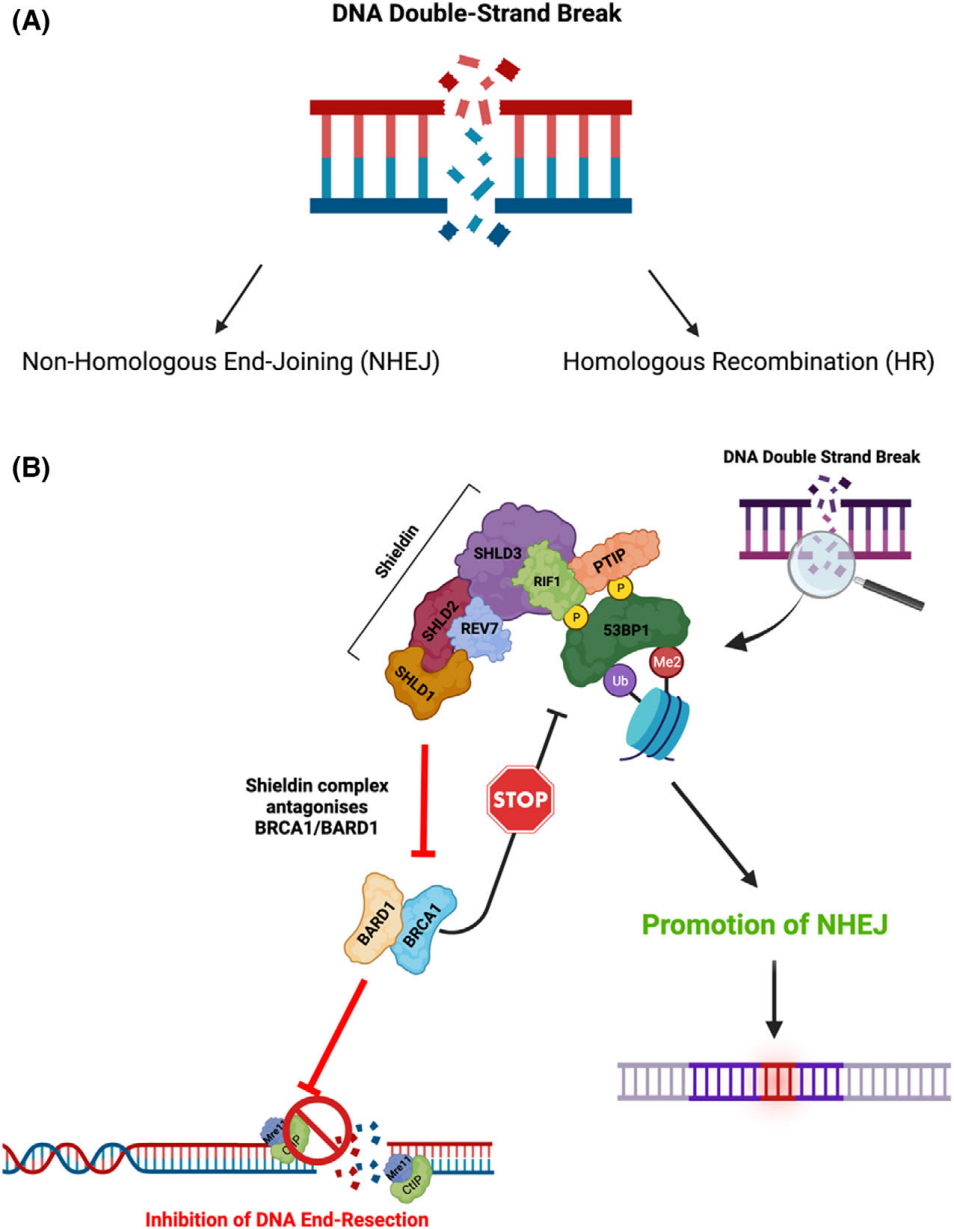
DNA double‐strand break‐end protection. (A) DNA DSBs are predominantly repaired by two major repair pathways: NHEJ and HR. (B) A schematic of the DNA DSB end protection by 53BP1‐RIF1‐REV7‐Shieldin complex. 53BP1 binds to nucleosomes modified by H4K20 methylation and H2K15 ubiquitylation. It is then phosphorylated in an ATM/ATR‐dependent manner, which facilitates the recruitment of downstream effectors, including RIF1 and PTIP. RIF1 interacts with the Shieldin complex (SHLD1, 2, 3 and Rev7), which partly functions to antagonize the disruption of the 53BP1‐nucleosome interaction by BRCA1/BARD1. This helps to block the initiation of DNA end resection by Mre11/CtIP, promoting the repair of DNA DSBs by NHEJ. Figure created using Biorender. [Colour figure can be viewed at wileyonlinelibrary.com]

DNA DSBs are extremely genotoxic events, and just one unrepaired DSB can result in cell death or in the rapid acquisition of mutations, leading to genome instability and the development of malignancy [72]. Mammalian cells have developed two major pathways to repair DNA DSBs, NHEJ and HR (see Box 1 and Fig. 4A). We refer readers to a recent extensive review on this topic for more information [120]. In brief, NHEJ involves ligating the broken DNA DSB ends with minimal processing of the break ends. As explained in Box 1, the DNA DSB is recognized by DNA‐binding proteins such as the Mre11‐Rad50‐NBS1 (MRN) complex, PARP, or the Ku70/Ku80 heterodimer. After the ends are protected and brought into proximity, as shown in Box 1, DNA ligase IV ligates the ends to repair the DNA. Given the minimal processing of the broken DNA ends and that religation occurs without a template, DSB repair by NHEJ increases the risk of genome instability because of the likelihood that deletions and insertions occur at the breakpoint. Despite this limitation, NHEJ plays a crucial role in promoting error‐free gene conversion at the expense of utilizing more mutagenic forms of repair, including single‐strand annealing (SSA) and alternative end joining (Alt‐EJ) [121]. NHEJ also plays crucial roles in V(D)J and class switch recombination, expanding the antibody repertoire of the immune system; in keeping with this, defects in NHEJ can result in immunodeficiency [122, 123].

By contrast, HR involves the exchange of equivalent DNA sequences between homologous or sister chromatids during S/G2 phases of the cell cycle and therefore has a higher fidelity relative to that of NHEJ. A prerequisite for HR is the generation of long stretches of ssDNA through the process of DNA end resection. DNA end resection is a complex, cell‐cycle‐regulated process that involves the removal of the 53BP1, Rev7, RIF‐1, and Shieldin complex from the DNA DSB site (see Box 1 and Fig. 4B). Several mechanisms for this removal have been proposed, including via Tip60‐mediated histone acetylation at Histone 4 lysine 16 (H4K16Ac) [124]. In recent years, arginine methylation of the essential RUVBL1 subunit of Tip60, mediated by PRMT5, and the alternative splicing of Tip60, has also been shown to dynamically regulate this process of 53BP1‐complex removal [125, 126]. Several BRCA1‐dependent mechanisms have also been described. One such mechanism involves BRCA1‐mediated PP4C‐dependent 53BP1 dephosphorylation, which results in the release of RIF1 from the 53BP1 complex, promoting end resection [127]. H2A ubiquitination mediated by the BRCA1/BARD1 complex has also been shown to recruit the chromatin remodeler SMARCAD1, which helps to reposition 53BP1 and to slide nucleosomes around the DNA DSB site facilitating long‐range DNA end resection [128]. Defects in DNA double‐strand break repair, in particular mutations in genes involved in HR, are associated with an increased risk of cancer, with *BRCA1/2* mutations in breast and ovarian cancers being one of the most common examples of this [129, 130].

### 3.7 Cell death

When cells experience DNA damage that is too severe to be repaired, programmed cell death occurs, most commonly by apoptosis. Apoptosis occurs under both physiological and pathological conditions. For example, apoptosis is a critical mediator of essential physiological events in mammals, including embryonic development, involution of the lactating breast, and shredding of the endometrium during the menstrual cycle [131]. However, apoptosis is also a frequent feature of pathological events, most notably cell death in response to physiological stimuli such as hypoxia, or in response to ionizing or UV irradiation, and in myocardial infarction and in neurodegenerative diseases, such as Parkinson's and Alzheimer's Disease [132].

Another crucially important type of cell death is mediated by PARP1 in response to high levels of oxidative DNA damage. First described by Yu et al. [133], this phenomenon is called *Parthanatos* in homage to *Thanatos*, the personification of death in Greek mythology [134]. In Parthanatos, PARP1 is activated following oxidative damage, and parylation (PAR) polymers subsequently form. Apoptosis‐inducing factor (AIF1) is then activated and released from the mitochondria to catalyze caspase‐independent cell death [133, 134, 135]. This process of programmed cell death has been implicated in a range of age‐related pathologies, most notably Parkinson's disease and Alzheimer's disease, and in age‐related macular degeneration, a progressive degenerative eye disease [136, 137]. Understanding key mechanistic events that underpin degenerative ageing conditions is of crucial importance for the development of therapeutic interventions. Indeed, given an ageing global population and the ever‐increasing economic burden of age‐related pathologies, developing treatments for Alzheimer's disease, Parkinson's disease, and other degenerative age‐related disorders remains a vitally important focus of ageing research.

### 3.8 Cell senescence

Cells with unrepaired DNA lesions that manage to avoid programmed cell death via apoptosis or Parthanatos often end up in a state of cellular senescence. Cellular senescence is a typically irreversible form of proliferative arrest that globally regulates cell fate and is widely considered to be a hallmark of ageing [138], and an emerging hallmark of cancer [64]. Cellular senescence can be triggered by many different stimuli, including but not limited to, telomere shortening, mitogenic signals, oncogene activation, and genotoxic stress caused by radiation, oxidation, or other genotoxic insults, such as chemotherapy treatment [138, 139]. It is also caused by epigenome fluctuations and by the disruption of chromatin structure, nutrient, or metabolic deficiency, and by tissue damage and inflammation [140]. In the context of DNA damage, excessive and/or persistent activation of DDR signaling can induce cellular senescence. Irreparable DNA damage, including even just a single unrepaired DNA DSB, has been shown to be sufficient to induce cellular senescence [72, 141].

Telomere shortening can also activate the DDR to result in cell‐cycle arrest and senescence. To this end, the end‐replication hypothesis postulates that somatic human cells are deficient in sufficient levels of the catalytic subunit of telomerase to maintain telomeres following a finite number of cell divisions [139, 142]. In the context of oncogene‐induced senescence, oncogene activation causes a hyperproliferative state, which results in widescale hyperactivation of origins of replication [98]. Under normal conditions, only a small proportion of all licensed origins undergo activation (or origin firing). In addition, those origins that are activated are not all activated at the same time, functioning as a backup mechanism to re‐start DNA replication following the stalling or slowing of the replication machinery. The coordination of origin activation is regulated by a replication timing program that is influenced by a multitude of factors, including chromatin structure [143], ongoing transcriptional programs, and the availability of essential replication factors [144, 145]. For example, early replicating domains are often associated with actively transcribed regions of open chromatin, whereas late replicating domains are frequently residing within heterochromatic regions [143, 146]. The overzealous origin firing in hyperproliferative oncogene‐activated cancer cells can result in stalled replication forks and in the accumulation of DNA damage, leading to cellular senescence or cell death [98].

One of the major outcomes of cellular senescence is the production of a bioactive secretome referred to as the senescence‐associated secretory phenotype (SASP), which involves the release of a wide‐ range of activated immune proteins, including chemokines, cytokines, and proteases, which are unique to their cell or tissue of origin [147]. The role of SASP in ageing‐related diseases and cancer has been extensively described in two recent reviews [147, 148]. In brief, current thinking in the field is that acute senescence might protect against cancer and help to limit the development of tissue fibrosis. In this context, the immune‐mediated clearance of senescent cells has been demonstrated to suppress tumor initiation [149] and contribute to tumor regression [150, 151]. However, long‐term senescence is believed to drive ageing‐related disorders. Indeed, the role of senescence in organismal ageing has received a lot of attention in recent years, following the demonstration by van Deursen and colleagues that the genetic ablation of senescence cells in mice leads to a striking increase in organismal lifespan [152]. As a result, targeting senescence has become an attractive proposition through so‐called ‘senotherapy’, which aims to specifically target senescent cells. Many laboratories and companies are currently investigating drugs that can target senescent cells (senolytics) [147, 148]. SASP is also involved in wound healing [153] and in cellular reprogramming in response to tissue damage [154]. However, SASP has context‐dependent effects as it has also been shown to promote tumorigenesis, induce epithelial to mesenchymal transition, and increase tumor vascularization/angiogenesis [155].

Chronic inflammation underpins many ageing‐related pathologies and is often referred to in this context as inflammaging [156]. Interestingly, senescent cells have been linked to inflammaging, as their removal reduces pro‐inflammatory cytokines in aged mice [152, 157]. There are likely to be many ways in which SASP contributes to tissue dysfunction and ageing‐related diseases. Some of the most likely scenarios, however, involve the secretion of extracellular matrix proteins, immune cells, and cytokines that can directly remodel localized tissue environments and impact the fate of different cell types in these tissue microenvironments [158]. A classic example is that of atherosclerotic plaques, which consist largely of senescent ‘foamy’ macrophages [159], and which reduce the lumen of blood vessels, in turn increasing the risk of the blood clot and cardiovascular disease—one of the most common diseases associated with ageing [160].

It has been proposed that the duration, and the severity, of DNA damage is a key determinant of whether cells apoptose or senesce. For example, Petrova et al. [161] have suggested that short‐term but major damage is likely to result in a more immediate apoptotic cellular response, whereas mild, long‐term damage is more likely to result in a state of cellular senescence, as opposed to programmed cell death. Sufficient or prolonged activation of the DDR involves the activation of the p53/p21^WAF1/CIP1^ tumor suppressor pathway, the major pathway responsible for inducing senescence in response to DNA damage [162]. Many different proteins are involved in the recognition of DNA damage lesions, as described earlier. Upon detecting DNA damage, these proteins stimulate a coordinated signaling network, which in turn activates additional mediators of the DDR. As mentioned above, the kinases ATM and ATR serve as master regulators of this response. Although DNA damage activates both ATM and ATR, these kinases have distinct specificities for types of DNA damage. For example, ATM is predominantly activated by the presence of DNA DSBs, whereas ATR is predominantly activated in response to genotoxic stress caused by problems with DNA replication [73]. Once at the site of DNA damage, ATM and ATR can further propagate DNA damage signaling through the phosphorylation of downstream mediators, such as the histone variant H2.AX to form γH2AX. γH2AX can then serve as a scaffold for the recruitment of other proteins into DNA damage‐induced nuclear foci, with the purpose of creating a hub of DNA repair proteins that congregate at the site of DNA damage [44, 73, 74, 163]. ATR and ATM also directly phosphorylate downstream substrates Chk1 and Chk2, respectively, which in turn phosphorylate the final effector proteins p53 and p21 to initiate cell‐cycle arrest and senescence.

In recent years it has become clear that there is a direct relationship between senescence and ageing. Indeed, analysis across multiple tissue types has demonstrated that the number of senescent cells increases exponentially with increasing chronological age [164]. In further support of this, clinical studies of childhood cancer survivors clearly demonstrate that chemotherapy and radiotherapy treatment results in long‐term side effects with clinical manifestations that are like those seen in ageing‐related pathologies, including organ dysfunction, secondary cancers, and impaired or degenerative cognitive function [165, 166]. This link between DNA‐damaging chemotherapy, senescence, and ageing‐like clinical manifestations has been further substantiated using *in vivo* preclinical models. Campisi and colleagues, for instance, developed a unique mouse, p16‐3MR, in which P16^INK4a^‐positive senescent cells can be tracked in live animals [167]. Senescent cells can also be ablated in this model via drug treatment. Using this model, Campisi and colleagues have shown that therapy‐induced senescent (TIS) cells persist and contribute to local and systemic inflammation. Moreover, elimination of this senescent cell population dramatically reduces several of the short and long‐term effects of drug treatment, including bone marrow suppression, cancer recurrence, and loss of physical strength. Strikingly, patients with increased expression of senescent markers in their T cells are significantly more likely to experience adverse effects from chemotherapy, most notably therapy fatigue and sickness [167]. This study, when combined with long‐term clinical follow‐up studies of cancer survivors, convincingly demonstrates the importance of senescent cells in the adverse long‐term effects of chemotherapy treatment and ageing‐like pathologies. Moreover, it supports the concept that targeting this senescent cell population could be an important therapeutic strategy for both the prevention of ageing‐related pathologies and for reducing the long‐term effects of chemotherapy treatment in cancer survivors.

### 3.9 Stem cell exhaustion

DNA damage is also linked to stem cell exhaustion during ageing, which occurs through a variety of processes, including apoptosis, premature differentiation, accumulating mutations, and cytostatic DNA‐damage checkpoint signaling [78]. A clear example of stem cell exhaustion is seen in hemopoietic stem cells (HSCs), which (like most adult stem cells) reside in a quiescent state that protects them from the genotoxic stress experienced during DNA replication [79]. This quiescent state, however, means that damaged DNA is more often repaired by less accurate DNA repair pathways, such as NHEJ (see Box 1), resulting in the accumulation of mutations at a much higher frequency. When mouse HSCs are forced out of quiescence and into a proliferative state, they show fewer mutational signatures following exposure to IR, compared with nonproliferative cells [168]. This suggests that a break from quiescence could be advantageous for DNA repair, enabling the activation of higher fidelity DNA repair mechanisms, such as HR [169]. In further support of the importance of DNA damage repair for stem cell maintenance, the key DDR kinase ATM is essential for HSC function. Indeed, *Atm* null mice exhibit impaired HSC function due to increased oxidative DNA damage and subsequently develop bone marrow failure [170]. Given the role of DNA damage in stem cell exhaustion, targeting the DDR to restore stem cell function has become an attractive therapeutic proposition. For example, the DePinho lab has demonstrated that the reactivation of telomerase in aged mice with dysfunctional telomeres and with increased DNA damage signaling can eliminate many of the associated degenerative phenotypes seen in these mice [168]. However, extreme care is needed when manipulating DNA damage signaling pathways in the context of stem cells, as many cancer cells manipulate these same pathways to overcome replicative senescence to drive malignancy [171]. It therefore remains to be seen whether targeting the DDR in stem cells is a viable option for the prevention or reversal of ageing phenotypes. In summary, mammalian cells have developed specialized and highly complex DNA repair pathways to deal with the myriad of genotoxic lesions experienced daily. Failure to accurately repair these lesions can result in cellular outcomes such as senescence and stem cell exhaustion, which as described above, can contribute to the organismal decline and premature ageing.

## 4 DNA damage and ageing‐related disease

Defects in DNA repair pathways that impact genome instability can have major impacts on organismal fitness and survival, which can lead to a range of diseases including cancer and neurodegeneration. In this section, we discuss how genome instability and defects in DNA repair can cause disease.

### 4.1 Genome instability and cancer

Genomic instability is a feature of most solid tumors and adult‐onset leukemias. It is characterized by abnormal chromosome number and/or structure and by changes to DNA in the form of nucleotide deletions, insertions, or substitutions [172]. Chromosome instability (CIN) has long been considered an important facilitator of tumorigenesis. This is supported by the many CIN mouse models that are either tumor prone, or that exhibit accelerated tumorigenesis when crossed onto sensitized genetic backgrounds [173]. Indeed, when these DNA changes occur in oncogenes or lead to the loss of tumor suppressor genes, they can result in selective cell growth and survival advantage, leading to uncontrolled cellular proliferation and malignancy [173]. Alterations to DNA copy number are also routinely observed across a range of tumor types, including amplification of the *EGFR* gene in gliomas [174], of *MYCN* in neuroblastoma [175], and of the *ERBB2* gene in breast [176] and ovarian cancer [177]. Conversely, loss of tumor suppressor genes such as *PTEN*, *p53*, *SIRT6*, and *VHL* is reported in a wide range of tumors [37, 178, 179, 180]. Focal amplification of chromosomal regions has also been shown to be important in cancer. For example, amplification of the chromosomal regions, chromosome 1q21.2 and chromosome 20q11.21, which harbor oncogenes or pro‐survival/chemoresistance genes, protects cancer cells from chemotherapy [181, 182]. The mechanisms by which these focal amplification events occur are still not fully understood. However, recent studies have described a transient mechanism by which site‐specific focal amplification or transient site‐specific copy gains (TSSGs) can occur [183, 184]. These TSSGs are driven by a network of histone lysine methyltransferases and demethylases, which regulate histone marks such as H3K27me3, H3K9me3, and H3K4me3 at specific genomic loci [185, 186]. H3K4me3 recruits the histone lysine demethylase KDM4A, which then interacts with components of the replication machinery at these loci, driving DNA re‐replication and focal extra‐chromosomal DNA amplification of regions that harbor pro‐survival genes and oncogenes, including *EGFR* [185, 186]. How these extrachromosomal DNA amplification events become permanently integrated into the genome remains the subject of intensive investigation, and whilst the mechanisms are not yet understood, it is tempting to speculate that the mis repair of DNA lesions that results from DNA re‐replication events could be a driver of these integration events. Consistent with this hypothesis, a recent study has demonstrated that chromothripsis—the catastrophic shattering of chromosomes and the religation of DNA fragments in random order—is a major driver of extrachromosomal DNA amplification events in cancer [187]. Therefore, it is highly plausible that DNA repair mechanisms are also involved in the subsequent integration and selection of these extrachromosomal DNA amplification events.

### 4.2 DNA repair defects and premature ageing syndromes

**Fig. 5:**
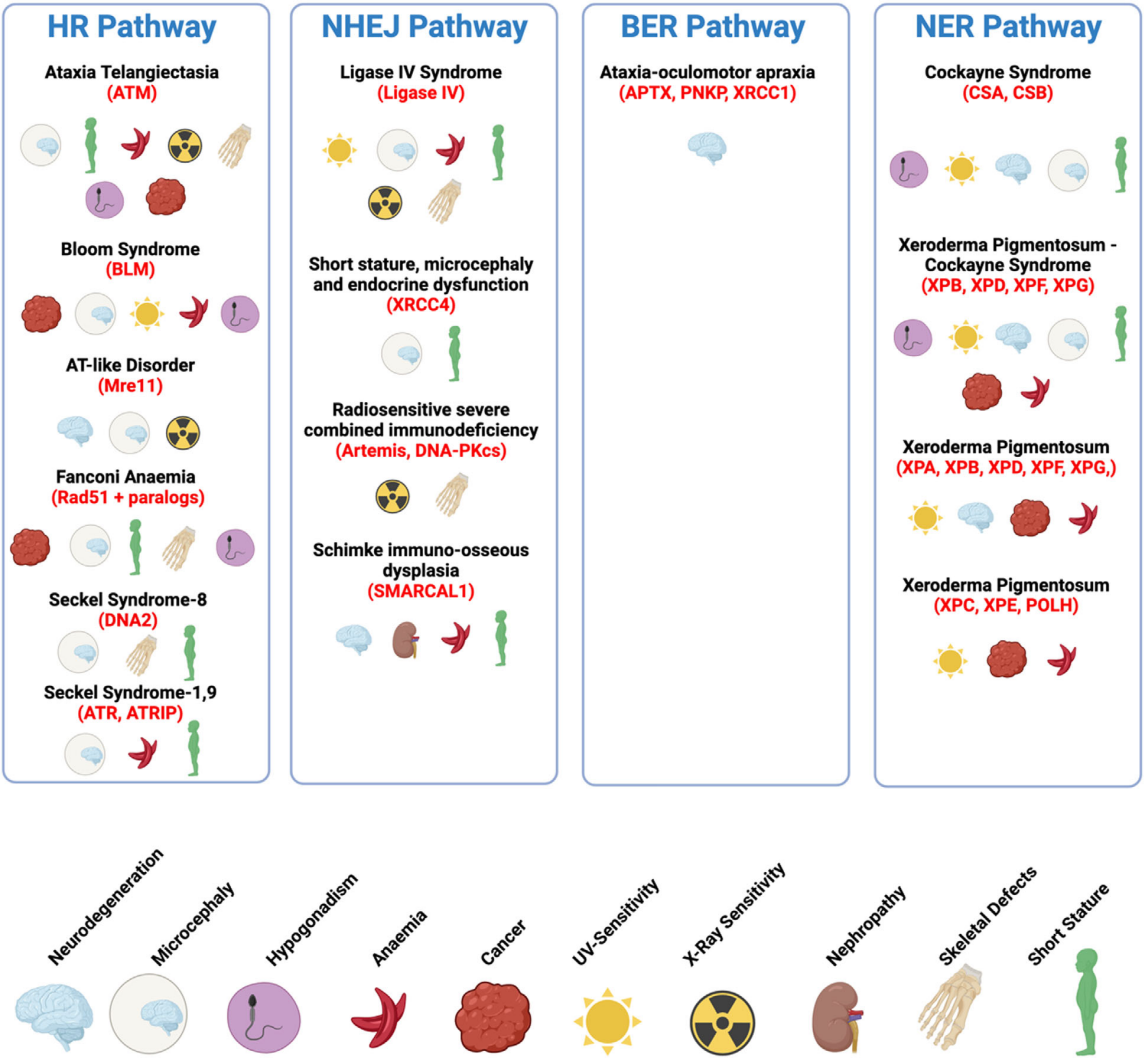
Premature ageing syndromes associated with defects in DNA repair. This schematic shows different human diseases that are associated with defects in the following DNA repair pathways: the HR pathway, the NHEJ pathway, the BER, and NER pathways. Mutations in genes encoding key components of these repair pathways (highlighted in red) and their reduced expression result in a range of progeroid/premature ageing syndromes and cancers. Also shown are the clinical features of these disorders, some of which are common to different defective DNA repair pathways. Figure created using Biorender. [Colour figure can be viewed at wileyonlinelibrary.com]

The critical importance of DNA repair for maintaining cell viability is further highlighted by the variety of human genetic syndromes underpinned by mutations or deletions in genes encoding essential components of the DDR. These human syndromes represent segmental progerias, which capture some but not all symptoms of ageing [188]. Examples of these syndromes include Cockayne syndrome (caused by mutations in the *ERCC6* and *ERCC8* genes), Ataxia‐ Telangiectasia (*ATM*), AT‐like disorder (*Mre11*), Bloom syndrome (*BLM*), Seckel syndrome (*DNA2*), Fanconi Anemia (which is characterized by defects in ICL repair), Werner syndrome (*WRN*), Meir‐Gorlin syndrome (*ORC1, ORC4, ORC6, CDT1, CDC6*) and Xeroderma Pigmentosa (*XPA‐G, XPV*) [3, 189]. A summary of some of the most common human syndromes caused by defects in DNA repair genes and their associated clinical features is provided in Fig. 5. Despite the spectrum of human syndromes caused by mutations in the many different genes that affect DDR signaling pathways, these syndromes share some common clinical features: immune deficiency, impaired growth/short stature, hypogonadism, intellectual and developmental delay, and microcephaly [3, 189]. Notably, many of these human syndromes exhibit evidence of accelerated ageing. For example, Bloom, Werners, and Rothmund‐Thomson syndromes (RTS) all result in progeroid syndromes with clinical features that range from early‐onset arteriosclerosis, premature hair graying, chronic obstructive pulmonary disease (COPD), osteoporosis, diabetes, and cancer [190]. In addition, patients with DNA repair/genome instability syndromes often exhibit hypersensitivity to X‐ray and UV exposure [191]. For example, patients with Xeroderma Pigmentosa (*XPA‐G, XPV* mutations), are extremely sensitive to neurodegeneration, and show premature photo‐ageing of the skin [81, 82]. Consistent with this, it is estimated that patients with Xeroderma Pigmentosa have a 1000‐fold increase in the risk of developing skin cancer [81, 82].

Similarly, patients with Ataxia‐Telangiectasia are extremely radiosensitive and exhibit significant premature neurodegeneration and onset of malignancy [192]. Therefore, efficient, and accurate repair of DNA damage is essential to prevent premature ageing and ageing‐related disease, including cancer.

### 4.3 DNA damage theory of ageing

The DNA damage theory of ageing states that unrepaired DNA damage causes genome instability and contributes to the ageing process [43]. Whilst the exact types of DNA lesions responsible for ageing are still debated, there is a wider view that genome stability and ageing prevention are prioritized at an early age via natural selection, when as a species we are in our reproductive prime. Indeed, as we age, our molecular fidelity declines along with our reproductive potential, and this is directly correlated with the importance of our individual survival for the long‐term maintenance of our species [43, 193]. In keeping with this premise, Preston et al. [194] used a DNA repair reporter system in *Drosophila* and demonstrated that the relative usage of specific DNA repair pathways changes as organisms age. For example, the relative usage of homology‐directed repair increased from 14% in the young male *Drosophila* germline to around 60% in aged males [194]. Given that homology‐directed repair can only occur in late S phase or the G2 phase of the cell cycle, it is tempting to speculate that one plausible mechanism for the accumulation of DNA damage and somatic mutations is this change in DNA repair pathway utilization. Supporting this idea, more recent work from Vera Gorbunova, as discussed above, demonstrated that DNA double‐strand break repair is more efficient in long‐lived species, by comparing repair efficiency between a panel of species with diverse lifespans [38]. Mechanistically they showed that the HDAC SIRT6, which as discussed above is associated with increased lifespan, was a major driver of this DSB efficiency, identifying specific mutations in at least five amino acids that drive this differential DNA repair activity across species [38]. The strength of the links between DNA repair and mechanisms of ageing is seemingly compelling enough for DNA damage to be considered as a primary cause of ageing. However, there is still some criticism of this notion [78]. Firstly, one could argue that if DNA repair is critical to the ageing process, improving DNA repair alone should increase lifespan, and to date, there is little direct evidence that this is possible. However, as elegantly described in a recent review by Schumacher et al. [78], this is a somewhat weak argument, given the enormous complexity of the DDR and the myriad of lesions that are repaired by hundreds, if not thousands, of proteins functioning across a range of interconnected pathways. Indeed, the idea that the simple upregulation of a handful of DNA repair genes can overcome this complexity in an ageing organism is perhaps too simplistic a proposition. Interestingly, however, a recent study performed whole‐genome sequencing in semi‐supercentenarians (105 years of age) and supercentenarians (110 years of age) showed significant enrichment for genes involved in DNA repair compared with younger, geographically matched controls. They also had a much lower mutational profile compared with healthy controls [195]. Whilst this study was performed on a relatively small sample size of approximately 400 participants across two independent cohorts, its findings are consistent with the notion that improved genome stability/DNA repair is likely to be an essential characteristic for longevity. It remains an open question, however, whether DNA repair mechanisms are more active in this long‐lived population.

Another key criticism of the idea that DNA damage is a direct cause of ageing is based on the idea that DNA damage and genome instability have wide‐ranging impacts that can lead to altered gene expression profiles, the slowing down or impeding of DNA replication, increased DNA replication, transcriptional stress, or increased senescence. As such, DNA damage is not a direct cause of ageing *per se*. While this is possible, the fact that numerous human instability syndromes/premature ageing syndromes are caused by mutations or deletions in DNA repair genes (as discussed above) argues for the direct role of DNA damage in ageing.

Further criticisms of the DNA damage theory of ageing are due to challenges associated with detecting spontaneous DNA damage in primary cells and tissues. Indeed, technological shortfalls limit the detection rate of DNA damage in primary cells and tissues, which translates to a low reproducibility between experiments/studies as a result. A recent review from Jan Vijg addresses these issues in exquisite detail, and we refer readers to this excellent article for a detailed discussion on this topic [196]. Briefly, studies have identified modest increases in spontaneous DNA damage in the livers of older mice when compared to younger mice, using both mass‐spectrometry and PCR‐ based analyses [197]. Interestingly, this DNA damage is less apparent in the brains of these mice, which makes it more difficult to directly link this DNA damage to ageing phenotypes that are commonly associated with declining brain function. More recent work has described time‐dependent delays in nascent RNA production in mice with defects in DNA repair, which could be rescued by a calorie‐restricted diet, implying a direct link between defective DNA repair, impaired transcription, and premature ageing [198].

Newer technologies including small nucleotide polymorphism (SNP) arrays and next‐generation sequencing demonstrated that genome mosaicism is widespread in human tissues, and this mosaicism is largely the result of somatic mutations [196]. Most studies to date in this area have been performed in blood, where somatic mutations often result in clonal hematopoiesis. How somatic mutations and clonal hematopoiesis drive ageing phenotypes is still the subject of extensive study. One study, however, has linked mutations in the epigenetic regulator Tet2 with atherosclerosis and heart disease—one of the most frequent diseases associated with ageing [199]. Mechanistically, deficiency of Tet2 in macrophages drives an increase in IL‐1β increasing the development of atherosclerosis in these mice [199].

As discussed by Jan Vijg in a recent review, most somatic mutations are unlikely to expand to clones that are readily detected by direct analysis in bulk tissues [196]. In now classic studies, Vijg and colleagues used unique mouse model reporter systems to assess somatic mutations associated with ageing [200, 201, 202]. These studies demonstrated that mutations occur in virtually all tissues and accumulate with age; however, there is huge variation in mutational frequency between tissues—the reasons for which are still unclear. These original experiments using mutations at specific reporter loci have limitations in that the representative mutational burden may not reflect that seen across the genome. Instead, newer methodologies are being used which take advantage of new genome‐wide sequencing techniques to begin to address this issue. The stochastic nature of somatic mutations and clonal expansion, however, means that much of this analysis will need to be done at single‐cell resolution, which remains a challenge in the field [196].

The exact mechanistic explanation as to how DNA damage and subsequent somatic mutation(s) drive ageing is still unclear. There is likely a fine balance between somatic mutation level and dramatically altered cell/organismal fitness. Therefore, it is reasonable to speculate that subtle changes occur over time, driven by genome instability‐induced somatic mutations. These mutations, which vary across individuals, may impact common biological pathways or gene regulatory networks, which ultimately lead to age‐related functional decline and disease patterns that are similar across individuals.

In summary, defects in genes involved in DNA repair and genome stability maintenance can cause segmental progeroid diseases. In addition, somatic mutations accumulate across tissues with age, which likely drives disruptions in key transcriptional programs, in addition to other key biological processes, culminating in the loss of fitness and organismal decline. Therefore, on balance, it seems that the proposition that DNA damage and genome instability are major drivers of ageing and ageing‐related disease, including cancer, is an increasingly convincing one.

## 5 DDR therapeutics in cancer

As we have discussed above, defects in DNA repair are associated with ageing‐related diseases including cancer and neurodegenerative disease. In the context of cancer, inflicting DNA damage has been the mainstay of therapeutic treatment for decades. In recent years, more specific targeting of DNA damage repair pathways has become a major focus in the development of novel cancer therapeutics, as a direct result of an increase in our understandings of the fundamental mechanisms of DNA repair. In this section, we will discuss some of these developments and their importance for ageing‐related disease.

### 5.1 PARP inhibitors

The vulnerabilities in cancer caused by increased levels of genome instability have been exploited for many decades in the clinic, using DNA‐damaging radiotherapy and chemotherapy. For example, agents such as cisplatin, carboplatin, oxaliplatin, and mitomycin C, which impede DNA replication by causing inter and intra‐strand DNA crosslinks, have been the mainstay of chemotherapeutic regimens for decades. In the late 1980s/early 1990s, Etoposide (Topoisomerase II inhibitor) and Irinotecan (Topoisomerase I inhibitor) were approved for use in treating colorectal cancer by the US Food and Drug Administration (US‐FDA), representing the first generation of targeted DNA‐damaging therapeutics [73, 172]. Whilst undoubtedly effective in certain clinical contexts, the prolonged and concentrated use of these agents is severely limited by the significant toxicity they cause to patients, largely due to collateral damage to healthy tissues following systemic treatment.

In the mid‐2000s, the development of poly (ADP‐ribose) polymerase (PARP) inhibitors signaled the next generation of targeted DDR therapeutics. PARP1 and PARP2 are members of the PARP protein superfamily that catalyze the parylation of target proteins using NAD+ as a substrate [203]. PARP family proteins play a crucial role in recognizing SSBs and DSBs and in helping to recruit the DNA repair machinery to repair DNA lesions; they also stabilize stalled replication forks during DNA repair [204]. The best‐understood role for PARP1, however, is in the repair of SSBs. As previously discussed, in the absence of PARP1 activity, SSB repair is impaired due to the collapse of the replication fork machinery during DNA replication. This results in extremely genotoxic DNA DSBs in the S phase of the cell cycle. In 2005, two seminal studies demonstrated that cancer cells deficient in *BRCA1* or *BRCA2* exhibit selective and exquisite sensitivity to PARP inhibition [129, 130]. These studies suggested that HR, which is the dominant pathway for the repair of S phase DNA DSBs, buffers the effect of PARP inhibition in normal cells, preventing its toxicity. However, in contexts whereby HR is impaired, such as in cells that have lost the *BRCA1* or *BRCA2* genes, this buffering does not occur, resulting in extreme PARP inhibition‐mediated toxicity.

The first PARP inhibitor to receive regulatory approval from the FDA was Olaparib in 2014, for the treatment of advanced‐stage *BRCA1/2*‐mutant ovarian cancers refractory to ≥ 3 prior lines of therapy [42, 204]. To date, there are now three different PARP inhibitors approved by the FDA for therapeutic use (Olaparib, Rucaparib, Niraparib) in a range of clinical settings, including *BRCA1/2* mutated ovarian and breast cancers, and most recently in metastatic germline BRCA mutant pancreatic adenocarcinoma that has not progressed following 16 weeks of first‐line, platinum‐based chemotherapy [42, 204]. The use of PARP inhibition is also gaining popularity as maintenance therapy, following a complete clinical or pathological response to platinum‐based chemotherapies. This is largely due to the improved toxicity profile of PARP inhibitors when used as a monotherapy compared with conventional platinum‐based agents [205]. When using DNA‐damaging therapy in patients with DDR‐defective tumors, it is critical to monitor the risk of secondary malignancies caused by increased genome instability in response to therapy. Encouragingly, the risk of secondary malignancies, such as acute myeloid leukemia or other myelodysplastic syndromes, appears to be relatively uncommon in patients treated with PARP inhibitors, indicating that PARP inhibitors can be used safely for longer‐term maintenance therapy [42].

Several clinical trials have also reported PARP inhibition to have beneficial therapeutic outcomes in tumors without known *BRCA1/2* mutations [206]. Unsurprisingly, efficacy has been reported in tumors with known homologous recombination deficiencies (HRD), as defined by loss of heterozygosity (LOH), large‐scale translocations (LSTs), and telomeric allelic imbalance (TAI). Somewhat surprisingly, however, efficacy has also been reported in breast and ovarian tumors with neither *BRCA1/2* mutations nor a known HRD signature [42, 206]. With this in mind, Yap and colleagues have recently proposed expanding the concept of ‘BRCAness’ beyond its original meaning of HR deficiency caused by *BRCA1/2* mutation. They instead propose the term HRDness, given the mounting evidence that HR deficient tumors are sensitive to PARP inhibitors in the absence of *BRCA1/2* mutations [42, 206]. Ultimately, it will be important to identify additional biomarkers that can predict sensitivity to PARP inhibition so that patients can be stratified to maximize the clinical benefits of PARP inhibitor monotherapy. Indeed, identifying additional factors involved in the HR pathway(s) and their contribution to the efficacy of PARP inhibitor therapy is currently a major focus in the DDR field.

The DNA lesion that is primarily responsible for genomic toxicity in response to PARP inhibition is also a matter of current debate. As described above, the inhibition or trapping of PARP1/2 onto DNA is believed to result in impaired SSB repair, replication fork collapse, and DNA DSBs that can only be repaired in HR‐proficient cells. However, this paradigm has been questioned with the suggestion that defects in replication gap suppression might cause PARP inhibition‐associated genotoxicity [207]. Indeed, recent studies have demonstrated that PARP inhibition in both mouse and human cells results in the accumulation of postreplicative ssDNA gaps, and that exposure to these ssDNA gaps is a key determinant of sensitivity to PARP inhibition [208, 209]. This replication gap suppression (RGS) model is thought to be more consistent with clinical data than the model that bases PARP inhibitor sensitivity on the inability to repair DNA DSBs. This is particularly so in the context of HR‐ and fork‐protection proficient cells that exhibit unexpected sensitivity to PARP inhibition [207, 210]. Additional preclinical and clinical data are needed to substantiate the RGS model of PARP inhibition‐induced genotoxicity. Recent studies make it entirely plausible that both DSB‐dependent and DSB‐independent mechanisms of PARP inhibitor sensitivity exist. Indeed, the RGS model is becoming increasingly hard to discount given the increasing evidence that links PARP‐inhibitor resistance to HR‐independent mechanisms [209, 211]. We therefore need to (a) identify additional HR mediators that govern PARP inhibition sensitivity, which could serve as prognostic biomarkers in the clinic; and (b) remain open to identifying additional factors that mediate PARP‐inhibitor sensitivity in a DSB/HR‐independent manner.

Identifying key proteins involved in DNA repair pathways that can alter therapeutic response is a major focus in the DDR field. Large‐scale siRNA‐based, and more recent genome‐wide CRISPR knockout, screens have been used for these studies. However, the complete knockout of many essential genes results in cell death, leaving such genes undetectable in these screening approaches. To circumvent this, our laboratory has recently optimized high‐throughput screening platforms coupled with cDNA expression libraries to enable the detection of novel chromatin factors involved in DNA repair [212]. Additional studies of this nature will help us to elucidate key mechanisms involved in DNA repair and will facilitate the development of novel therapeutic approaches for a range of age‐related pathologies underpinned by genome instability.

### 5.2 Acquired PARP inhibitor resistance

Despite promising clinical responses to initial PARP inhibitor treatment, particularly in ovarian cancer, it is now apparent that most patients experience *de novo* or acquired resistance to this therapy [213]. A major focus of the DNA repair field is to understand the underlying mechanisms that underpin this therapeutic resistance. To date, several different mechanisms have been discovered using both tumor biopsies and preclinical models, which have shed light at the molecular level on how PARP inhibitor resistance can develop in the clinic [213].

One of the most common causes of PARP inhibitor resistance in the clinic is the development of secondary mutations. These mutations revert a defective HR gene (such as mutated *BRCA1, BRCA2, RAD51*, or *PALB2*) to its wild‐type sequence, restoring the cellular capacity for HR. Indeed, it is estimated that this could be the mechanism of resistance in as many as 20% of ovarian cancer patients who develop PARP inhibitor resistance [214]. HR restoration is also a key mechanism of PARP inhibitor resistance identified in several preclinical models. For example, loss of 53BP1 in BRCA1 null mouse and human cells induces resistance to PARP inhibition [215]. From a mechanistic viewpoint, this is entirely consistent with the antagonism that occurs between BRCA1 and 53BP1 for DNA end resection, which is a prerequisite for HR [216]. Moreover, the loss of other 53BP1 complex components that inhibit DNA end resection (e.g., REV7, RIF1) [217, 218], and that of the recently described Shieldin complex, also restore HR and cause PARP inhibitor resistance [51, 52, 53].

Genome‐wide CRISPR knockout (KO) screens are being performed by many labs worldwide in order to identify the potential mechanisms of PARP inhibitor resistance in different cancer contexts. For example, Chowdhury and colleagues have utilized a CRISPR‐Cas9 KO library to identify genes whose loss confers resistance to clinical PARP inhibition and to platinum‐based chemotherapy agents in *BRCA1* null patient‐derived ovarian cancer cell lines [219]. From their genetic KO screen, they identified Dynein light chain 1 (DYNLL1) loss as a major driver of platinum resistance and of PARP inhibitor resistance in *BRCA1* null ovarian cancer cell lines. Mechanistically, DYNLL1 limits the nucleolytic degradation of DNA ends by interacting with the MRN complex and with BLM helicase and DNA2 endonuclease. Thus, DYNLL1 serves as a critical anti‐resection factor that directly influences response to DNA‐ damaging chemotherapy [219]. It is becoming clear that the misregulation of DNA end resection via a range of different mechanisms can influence the response to DNA damaging chemotherapeutics, including PARP inhibition. Replication fork stabilization is also a major driver of PARP inhibitor resistance in preclinical models [220]. In addition, mutations and/or the reduction in PARP1 protein expression can drive resistance to PARP inhibitor therapy [221], while the stabilization of BRCA1 isoforms is also a driver of PARP inhibition resistance. In this context, an HSP90‐dependent mechanism has been described, leading to the investigation of HSP90 inhibition as a novel approach to restoring PARP inhibitor sensitivity in this context [222, 223]. It will be interesting to see in the coming years, the frequency with which resistance mechanisms seen in preclinical models appear in the clinic. Nonetheless, understanding the fundamental basic science mechanisms of DDR therapeutic resistance is essential for developing alternative clinical approaches for prolonging the lives of patients with cancer.

### 5.3 PARP inhibitors and ageing

In addition to its direct role in impacting DNA repair as a cancer therapy, there is also increasing interest in the role of PARP inhibition as a possible anti‐ageing therapy. PARP hyperactivation has been shown to lead to mitochondrial dysfunction, a common feature in neurodegeneration and ageing [224]. Indeed, PARP hyperactivation was shown to decrease activation of the NAD^+^‐ SIRT1‐PGC1α axis in cells from patients with Xeroderma Pigmentosum group A (XPA), a human disorder characterized by defective NER. Importantly, this mitochondrial function was restored by supplementation with NAD+ or PARP inhibition, which determined the rate of NAD^+^ consumption via hyperactive PARP1 as a driver of the disease [224]. Sirtuin activity has been associated with increased longevity in a range of different species (above), and there is increasing interest in the possibility of modulating sirtuin activity, in particular SIRT1, via PARP inhibition, in diseases associated with ageing such as heart disease [225] and neurodegenerative disease, including Alzheimer's disease [226]. It remains to be seen, however, how safe it would be to treat ageing‐related disease via inhibition of DNA repair machinery, especially in the context of an ageing, increasingly genomically unstable genome.

### 5.4 Synthetic lethality and drug discovery

The development of PARP inhibitors is an early, promising exemplar of how synthetic lethal relationships in the DDR can be exploited in targeted cancer therapy. Synthetic lethality is a genetic concept first described by Dobzhanksy in the 1940s and later in the 1960s by Lucchesi [227, 228]. It explains how a defect in one cellular pathway alone is insufficient to induce impaired cellular proliferation or survival; when combined with defects in additional pathways, however, these initial defects become lethal.

Following the clinical success of PARP inhibitors, there is now a major effort in the DDR field to identify additional synthetic lethal targets for novel cancer therapeutics. Indeed, work from the Durocher laboratory recently identified CIP2A as a synthetic lethal target in *BRCA*‐deficient cancer [229]. These authors performed a genome‐wide CRISPR‐Cas9 synthetic lethal screen to identify genetic vulnerabilities in *BRCA1/2*‐deficient cancer cells. They showed that CIP2A is essential for preventing the lethal mis‐segregation of acentric chromosomes by interacting with a critical DNA repair protein, TOPBP1. When they disrupted the CIP2A‐TOPBP1 complex pharmacologically, it induced lethality in *BRCA1/2* deficient cells, akin to *CIP2A* deletion. Thus, this study identifies a synthetic lethal therapeutic target in *BRCA*‐deficient cancers that is independent of HR or PARP inhibition. Further studies of this nature will identify additional therapeutic opportunities to induce synthetic lethality in tumors that are deficient/defective in DNA repair beyond PARP inhibition, considerably increasing the number of targeted therapeutic options for the treatment of cancer [230].

### 5.5 Emerging DDR therapies in cancer

Beyond PARP inhibition, many other inhibitors of DNA repair and DNA replication proteins are in various stages of clinical trials, as elegantly and extensively reviewed recently by Yap and colleagues [42, 206]. Whilst some of these emerging therapies will likely exhibit at least partial initial success, it is becoming increasingly apparent that much work remains to be done to optimize the efficient use of DDR‐targeted therapeutics for cancer treatment. Indeed, developing predictive biomarker assays, and better understanding the mechanisms of intrinsic and acquired therapy resistance, is of crucial importance for deploying these emerging therapeutics in the clinic for optimal patient benefit. It is also essential that we understand how combined treatments of DDR therapeutics can be tolerated by patients, and whether combining these treatments with other emerging novel cancer therapeutics (such as immune checkpoint blockade) might improve the clinical efficacy of these targeted therapies.

The targeting of DDR components might have therapeutic potential beyond the direct targeting of cancer cells. Indeed, a recent paper from the Patel lab suggests the DDR could also be targeted to prevent the classic features of late‐stage cancer and/or ageing [231]. In this study, transcriptional stress was investigated in the context of the human instability disorder, Cockayne syndrome. A major challenge in this field is that mutations of the Cockayne syndrome‐associated genes, *ERCC8* and *ERCC6*, do not recapitulate features of the human disorder in mice [232, 233, 234]. In addition, the endogenous factor(s) that cause the DNA damage that leads to the stalling of RNA Polymerase II (RNA Pol II) and the subsequent transcriptional stress are unknown [235, 236]. In this study, the authors identified endogenous formaldehyde as the cause of transcriptional stress and demonstrated that mice with deletions in the *adh5* and *Csb* (*Adh5* ^*−/−*^ *Csb* ^*m/m*^) genes exhibited features akin to those seen in human Cockayne syndrome, including cachexia and severe kidney failure. RNA‐sequencing of the nephron revealed that DNA damage/transcriptional stress caused damage to a subset of proximal tube cells that express the factor GDF15 [231]. Remarkably, treatment of *Adh5* ^*−/−*^ *Csb* ^*m/m*^ mice with an anti‐GDF15 antibody completely prevented DNA damage/GDF15‐ induced cachexia. The authors speculate that GDF15 inhibition might be a potential tool for preventing the nephrotoxic effects of chemotherapeutic agents, such as Cisplatin, and extending its possible use in patients with Cockayne syndrome to prevent kidney failure caused by formaldehyde‐induced Pol II‐mediated transcriptional stress [231]. Whilst more work is needed to address these possibilities, this work signals the dawn of a new era for DDR therapeutics in the context of cancer therapy. This new era is distinct from the direct targeting of cancer cells because it instead aims to inhibit or dampen down collateral DNA damage/toxicity in distant tissues and organs. Indeed, this potential future use of DDR therapeutics could be an effective tool with which to reduce toxicity in patients undergoing combination treatment and it could thus increase the feasibility of a range of combinatorial therapies across cancer types.

## 6 Concluding remarks

Ageing can be defined as the progressive decline in fitness and organismal function that ultimately brings life to an end. With improvements in living conditions and healthcare, humans are living longer than ever. Despite this vast improvement in human life expectancy, there is still an urgency to identify strategies to further maximize the human lifespan. As discussed in this review, pharmacological and nutritional supplementation is an attractive proposition in this regard [237]. The longevity and anti‐ageing field have become increasingly controversial in recent years, with the sirtuin family of proteins serving as an example of the many claims and counterclaims made as to the importance of certain proteins, and their pharmacological manipulation as the basis of increasing lifespan [238]. Presently, it is difficult to reconcile this literature with complete certainty, given the many different experimental models and experimental approaches used across many different laboratories. Indeed, one could argue that a major reason for this disparity is due to the inherent complexity of ageing, with genetic and environmental factors playing important roles and to different extents across different species. Nonetheless, the likelihood of identifying a linear and singular gene or family of genes solely responsible for organismal ageing seems increasingly remote.

With ever‐more increasingly sophisticated sequencing‐based technologies, our understanding of the range and frequency of somatic mutations across tissue types as organisms age is becoming increasingly apparent. The exact mechanisms that drive ageing in response to these somatic mutations are still unclear; however, it seems plausible that in the coming years, improvements and accessibility to single‐cell genomic and transcriptomic data across the different cell and tissue types will begin to uncover some of the key mechanistic drivers of ageing in tissue‐specific contexts, which will be vital for understanding many ageing‐related diseases. These approaches will undoubtedly give rise to a plethora of novel therapeutic strategies to treat a range of ageing‐related conditions and diseases.

Ultimately, maintaining genome stability is of crucial importance for the prevention of premature ageing and of ageing‐related diseases, such as cancer. Understanding how the cellular response to DNA damage balances the need for genetic diversity with that of genome stability will be needed to truly appreciate how long‐lived species survive and thrive. In the coming years, it will also be important to identify clinical biomarkers that can be used to stratify human patients for the optimal use of DDR‐targeted therapeutics both in the context of cancer and other ageing‐related disorders. In the context of cancer, understanding the mechanisms of resistance to DDR‐targeted therapies will be key for developing alternative therapeutic strategies, with the aim of making cancer a chronic, long‐term health condition. Elucidating the molecular mechanisms of DNA repair and genome stability maintenance in ever‐greater detail will undoubtedly move us closer to achieving this ambitious goal and continue to push the boundaries of the human lifespan.

## Conflict of interest

The authors declare no conflict of interest.
